# Fracture resistance of endodontically treated maxillary premolars restored with different bi-layered core and occlusal overlay materials: an in vitro study

**DOI:** 10.1186/s12903-026-09693-6

**Published:** 2026-09-15

**Authors:** Samar Saleh Mahmoud, Khaled Aly Nour, Omaima Hassan Ghallab, Khaled Mohamed Adel

**Affiliations:** Operative Dentistry Department, Faculty of Dentistry, Ain Shams University, Cairo, Egypt

**Keywords:** Fracture resistance, Core material, Overlay restorations, Endodontically treated teeth, Maxillary premolars

## Abstract

**Objectives:**

This study evaluated the reinforcing effect of various core and overlay materials used in bi-layered restorations on the fracture resistance of compromised endodontically treated maxillary premolars with mesio-occluso-distal (MOD) cavities.

**Methods:**

Three core materials were assessed: a packable short fiber-reinforced composite (everX Posterior (EXP)), its flowable counterpart (everX Flow (EXF)), and a dual-cure core material (GRADIA CORE (GC)). Each core material was combined with one of three overlay restorations: an indirect overlay (ID) using CERASMART 270, a direct overlay (D), or a semi-direct overlay (SD) using Gænial A’CHORD. A total of 110 sound maxillary premolars were allocated to 11 groups (*n* = 10), including a positive (sound teeth) and a negative (endodontically treated teeth restored with direct composite, without cusp reduction) control groups. Standardized endodontic treatment and MOD cavity preparation were performed with 2 mm buccal and palatal cusp reduction. Fracture resistance was assessed using a universal testing machine at a crosshead speed of 1 mm/min. Failure modes were analyzed using Firth’s logistic regression model, while fracture resistance was evaluated using two-way ANOVA followed by post hoc tests. The control groups were compared using an independent t-test. Statistical significance was set at *p* < 0.05.

**Results:**

The overlay restoration, Core material, and their interactions significantly influenced fracture resistance (*p* = 0.043, *p* < 0.001, and *p* < 0.001, respectively). Within the direct overlay group, EXP exhibited the highest fracture resistance (1124.61 ± 213.32 N), significantly higher than EXF but not significantly different from GC. EXP also demonstrated relatively high fracture resistance across the tested overlay restorations. EXF exhibited the lowest fracture resistance in the direct overlay group (596.14 ± 171.40); however, in the indirect overlay group, both EXF and GC showed significantly higher values than EXP. No significant differences among core materials were observed in the semi-direct overlay group. Unfavorable failure mode predominated, with no significant differences observed among the experimental groups.

**Conclusions:**

The fracture resistance of bi-layered restorations was significantly influenced by both the core and overlay materials and their interaction. EXP maintained high fracture resistance across various bi-layered restorations, whereas EXF demonstrated technique-dependent performance. The indirect overlay group achieved high fracture resistance, but was not superior to all direct overlay restorations.

**Clinical relevance:**

Packable short fiber-reinforced composite, particularly everX Posterior, demonstrated a promising synergistic effect with direct overlay restorations, resulting in fracture resistance comparable to that achieved with high-performance indirect overlay restorations.

## Introduction

Structurally compromised endodontically treated teeth exhibit a reduced ability to withstand occlusal forces and functional stresses. Consequently, restorative approaches should reinforce remaining tooth structure and promote favorable stress distribution during function to minimize the risk of further structural failure [1, 2]. Traditionally, full coronal coverage was recommended to protect such teeth. However, advancements in adhesive dentistry have enabled more conservative restorative approaches, including direct, indirect, and semi-direct restorations using materials such as particulate-filled composite (PFC), indirect ceramics, and hybrid ceramics [3]. Large direct PFC restorations may present a technique-sensitive approach, requiring meticulous incremental layering to minimize void formation. Moreover, polymerization shrinkage stresses, gap formation, cusp deflection, and enamel microcracking are common drawbacks associated with direct composite restorations [4]. Conversely, indirect ceramic and composite restorations offer favorable mechanical properties and controlled polymerization shrinkage; however, their clinical success primarily relies on proper design selection [5].

A comprehensive recognition of the inherent limitations of each restorative material has refined their clinical application, restricting their use to replace specific dental tissues rather than the entire lost tooth structure [6]. This concept led to the evolution of the biomimetic approach, which advocates restoring each dental tissue with material that closely mimics its natural properties [7]. This strategy promotes more harmonious biomechanical behavior within the tooth–restoration complex, thereby reducing stress concentration at the interface [8].

Bi-layered overlay restorations represent a promising biomimetic strategy [9]. This technique employs a bulk-fill core material to replace the lost dentin structure and an overlay restoration to restore the occlusal enamel structure. Dual-cured composites are widely used as core build-up materials. Their dual polymerization mechanism, combining light- and self-curing mechanisms, may facilitate a high degree of conversion, particularly in deeper areas. These materials offer favorable handling characteristics and high mechanical properties depending on their formulation, filler type, and filler content [10, 11]. However, void incorporation may occur during placement, potentially compromising their mechanical performance. On the other hand, fiber-reinforced composite (FRC) materials, such as short randomly oriented E-glass fiber-reinforced composites (SFRCs), leverage the reinforcing properties of fibers and can be used as dentin-substituting material [12]. The distinctive properties of fibers allow them to absorb stresses along their longitudinal axis [13, 14]. Furthermore, SFRCs may enhance fracture toughness by ceasing crack propagation, redirecting the crack pathway, or blunting the crack tips. Another determining factor reported by Garoushi et al. [15] was the thickness of the SFRC layer, which may enhance its rigidity as a subsurface structure and contribute to reinforcement of the occlusal restoration.

Occlusal overlay restorations offer more favorable stress distribution across a wider surface area compared with inlay restorations [16]. Direct overlay restorations with PFC have been reported to be more susceptible to fractures and may result in catastrophic failure under heavy loading [13, 17], whereas indirect composite blocks have demonstrated superior effect on stress absorption and resilience, as well as facilitating relatively simple repair protocols compared with glass ceramics [18, 19]. Nevertheless, studies have reported comparable mechanical performance between direct composite and indirect hybrid CAD/CAM occlusal restorations [3, 20, 21]. Other studies have suggested that incorporating a subsurface reinforcing composite material significantly improves the fracture resistance of structurally compromised teeth [12, 22].

Previous research studies have compared various bi-layered restorations; however, these studies have often focused on the effects of core and overlay materials independently. Therefore, the present study investigated the fracture resistance considering the effects and potential interaction of these two variables. The null hypotheses tested were: (1) there is no significant difference in fracture resistance among bi-layered restorations with different core build-up materials; (2) there is no significant difference in fracture resistance among bi-layered restorations with different overlay materials; (3) there is no significant interaction between the core and occlusal overlay materials with respect to fracture resistance; and (4) there is no significant difference in the fracture mode among different bi-layered restorations.

## Materials and methods

This study utilized three core materials: everX Posterior (EXP), everX Flow (EXF), and GRADIA CORE (GC). Two materials were used for the occlusal overlay: CERASMART 270 for the indirect overlay (ID) and G-ænial A’CHORD for both the direct (D) and the semi-direct (SD) overlays. Detailed information regarding material, their specifications, constituents, manufacturers, and lot numbers is presented in Table 1.

**Table 1:** Material, specifications, constituents, manufacturers, and lot numbers

Material	Specifications	Constituents	Manufacturer	Lot number
everX Posterior ™	Light-cured, packable SFRC	Bis-GMA, TEGDMA, short E-glass fiber 5–15 wt%, barium glass, silicon dioxide 61–75 wt%.	GC Corp, Tokyo, Japan	2,306,061
everX Flow ™ Bulk shade	Light-cured, flowable SFRC	Bis-MEPP, TEGDMA, UDMA, short E-glass fiber 25 wt%, barium glass 42–52 wt%.	GC Corp, Tokyo, Japan	2,310,181
GRADIA CORE ™ Universal shade	Dual-cure Core build-up	UDMA, NPGDMA, GDMA, TEGDMA, silanated Al-F-silicate glass, amorphous SiO_2_, TiO_2_, Fe_2_O_3_, MgO, initiator, accelerator, filler 75 wt%.	GC Corp, Tokyo, Japan	2,306,131
G-ænial A’CHORD ™	Packable light-cured nano-hybrid composite material, shade A_2_	Barium glass, DMA, silicon dioxide, initiator, pigment, stabilizer.	GC Corp, Tokyo, Japan	2,308,023
CERASMART 270 ™	Hybrid CAD/CAM block, shade A_2_ HT	Bis-MEPP, UDMA, DMA. Silica, barium glass 71 wt%.	GC Corp, Tokyo, Japan	2,306,266
G-CEM ONE ™	Universal self-adhesive resin cement, translucent shade	**Paste A**: UDMA, DMA, 10-MDP, Al-F-silicate glass, SiO_2_, initiator. **Paste B**: UDMA, DMA, Al-F-silicate glass, accelerator, pigment.	GC Corp, Tokyo, Japan	2,306,261
G2-BOND Universal™	Two-step universal adhesive	**Primer**: 4-MET, 10-MDP, 10-MDTP, DMA, acetone, water, initiators, fillers **Adhesive**: DMA, Bis-GMA, filler, photo-initiator.	GC Corp, Tokyo, Japan	2,305,181

*CAD/CAM* computer-aided design/computer-aided manufacturing, *10-MDP* 10-methacryloyloxydecyl dihydrogen phosphate, *4-MET* 4-methacryloyloxyethyl trimellitic acid, *10-MDTP* 10-methacryloyloxydecyl dihydrogen thiophosphate, *DMA* N, N-dimethylacrylamide, *Bis-MEPP* bis p-methacryloxy (ethoxy)1–2 phenyl-propane, *NPGDMA* neopentyl glycol dimethacrylate, *GDMA* glycol dimethacrylate, *Bis-GMA* bisphenol A glycidyl methacrylate, *Bis-EMA* ethoxylated bisphenol A dimethacrylate, *TEGDMA* triethylene glycol dimethacrylate, *UDMA* urethane dimethacrylate

### Sample size calculation

Sample size calculation was performed using G*Power version 3.1.9.7 software (Heinrich Heine University, Düsseldorf, Germany) based on data from a previous study [23]. The analysis was conducted using the F-test family and statistical test set as ANOVA: Fixed effects, special, main effects, and interactions. The analysis was based on the 9 experimental groups, with an interaction numerator degree of freedom of 4, a significance level (α) of 0.05, power (1-β) of 0.90, and an effect size (Cohen’s *f*) of 0.60 [24]. The resulting minimum required sample size was 6 specimens per experimental group. The sample size was subsequently increased to 10 specimens per group to ensure equal allocation, enhance statistical reliability, and account for biological variability. Thus, a total of 90 maxillary premolars were required for the experimental groups; 2 additional control groups (positive and negative) were included for comparison but excluded from the sample size calculation as they were analyzed separately.

### Teeth selection

This study received approval from the Ethics Committee of the Faculty of Dentistry, Ain Shams University (reference number: FDASU-RecE032145). A total of 110 sound maxillary premolars were collected from the Department of Oral Surgery, Faculty of Dentistry, Ain Shams University. The teeth were extracted for periodontal or orthodontic reasons. They were rinsed thoroughly, and root scaling was performed using a hand sickle scaler to remove residual attached tissue. They were visually inspected under 2.5x magnification (Ergo-vision, China), for cracks, wear, developmental defects, or caries. The teeth dimensions were measured for standardization with a digital caliper (Total TMT321501, Total Co, China) with an accuracy of 0.01 mm. The maximum accepted deviation range was ± 0.5 mm between teeth [25]. The dimensional criteria of selected teeth were as follows:

- Bucco-palatal dimension: 9.5 ± 0.5 mm, measured at the height of contour bucco-palatally.
- Mesio-distal dimension: 7.5 ± 0.5 mm, measured at the level of the proximal marginal ridge.
- Inter-cuspal distance: 6.0 ± 0.5 mm, measured between the buccal and palatal cusp tips.
- Crown height measured from the cemento-enamel junction (CEJ) to the corresponding cusp tip or marginal ridge: 8.5 ± 0.5 mm buccally, 7.5 ± 0.5 mm palatally, and 5 ± 0.5 mm proximally.
- Root length: 13.5 ± 0.5 mm.

The roots were then embedded in cold-cure acrylic resin molds (Acrostone Medical & Dental Supplies, Egypt) to a level 3 mm apical to the CEJ, with the tooth’s long axes positioned perpendicular to the molds. The specimens were then stored in distilled water containing 0.1% thymol solution (Thymol Crystals, ACG Alfa Chemical Group, Egypt) at room temperature.

### Specimen grouping and preparation

**Fig. 1:**
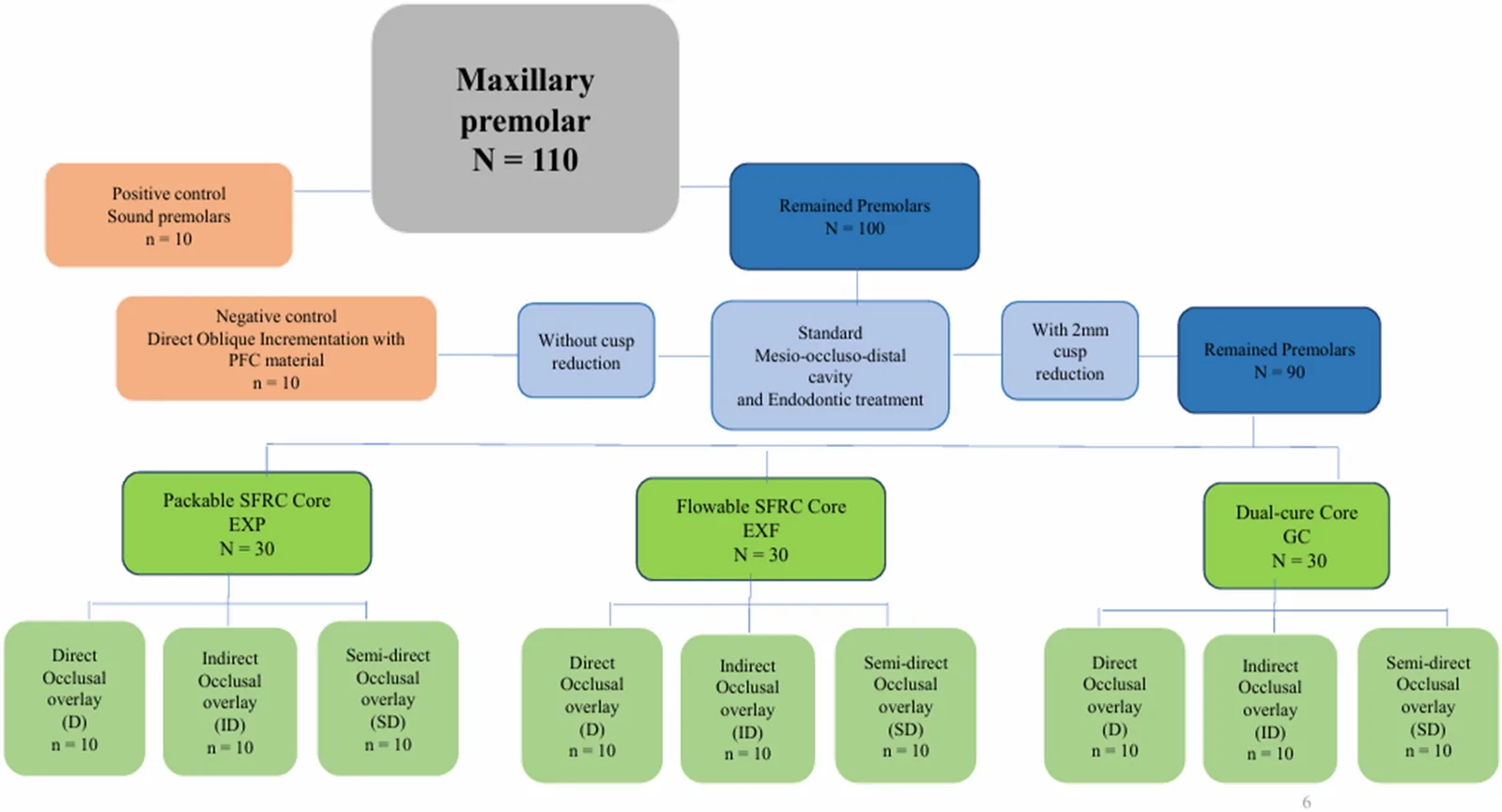
Illustrates the study design

The teeth were randomly allocated into 11 groups (*n* = 10), as presented in Fig. 1. The positive control group (PC) consisted of sound, unprepared teeth. The remaining specimens were allocated to 9 experimental groups according to the core and overlay materials, in addition to a negative control group (NC).

#### Pre-operative occlusal and proximal records

**Fig. 2:**
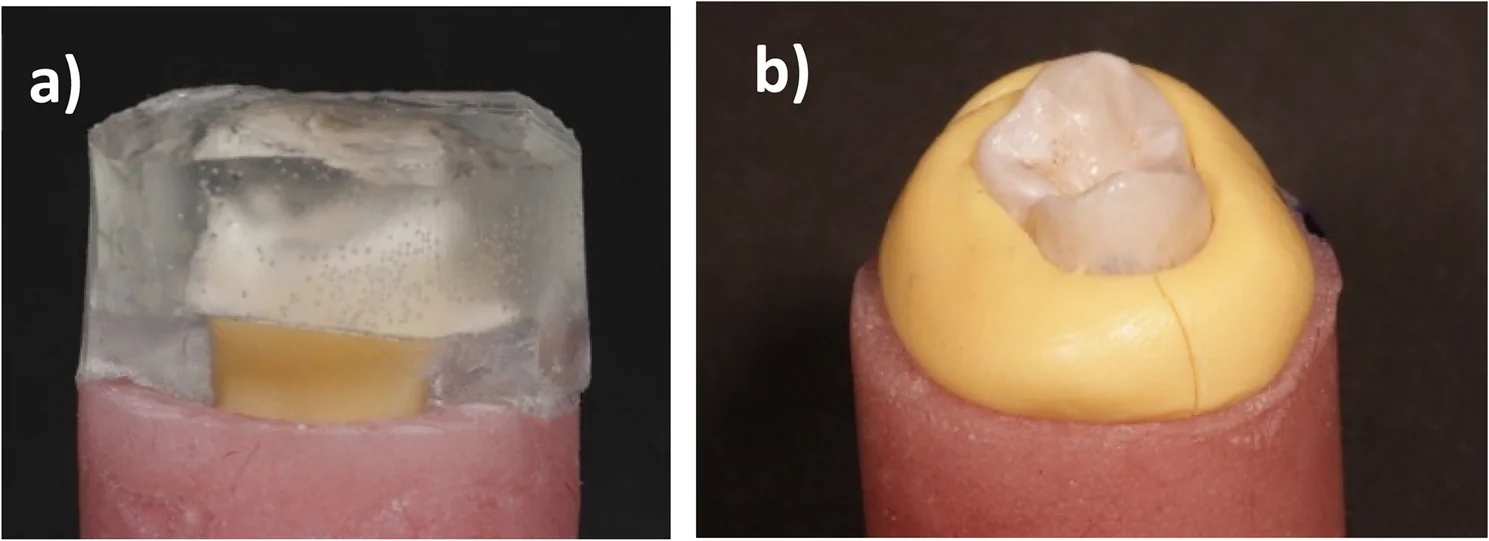
Pre-operative records: **a** Occlusal index from proximal view, showing the proximal vent and buccal and palatal parts acting as a pressure-resistance stopper. **b** Proximal index

A clear silicone material (EXACLEAR, GC Corp, Tokyo, Japan) was used to record the occlusal surface anatomy for the direct and semi-direct overlay groups. A proximal vent was created using a surgical blade to allow excess material to flow during seating. For the indirect overlay groups, the occlusal surfaces were digitally scanned before preparation. The proximal surface of all specimens was recorded using an addition silicone putty rubber base index (Zhermack Elite HD, Italy) at a level 2 mm apical to the marginal ridge (Fig. 2). 

#### Cavity preparation

**Fig. 3:**
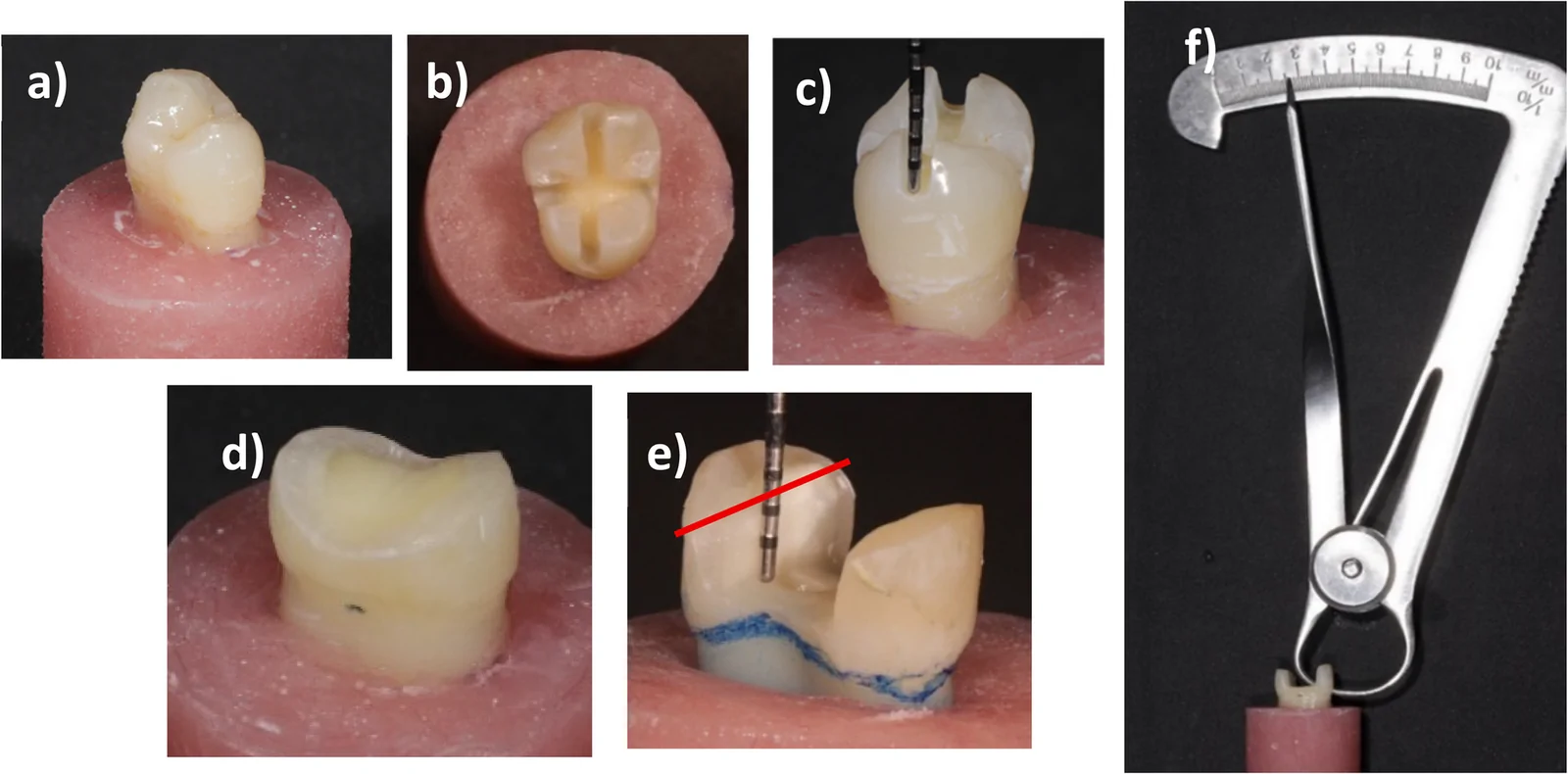
Steps of cavity preparation. **a** Tooth embedded in acrylic mold. **b** Mesio-distal and bucco-palatal depth cuts. **c** 2 mm depth cuts. **d** Specimen after buccal and palatal cusp reduction. **e** Standard MOD cavity with 2.5 mm depth; red line indicates the internal cavity margin. **f** Remaining wall thickness 2.5 mm

All cavity preparations were performed by a single operator. The negative control group (NC) received standardized mesio-occluso-distal (MOD) cavities without cusp reduction. The remaining premolars underwent 2 mm occlusal reduction using a high-speed handpiece (T3 line, Dentsply Sirona, PA, USA). Depth cuts were performed using a depth marker (FG 707 C, Intensiv, SA, Lugano, Switzerland), followed by cusp reduction using a parallel-sided, round-ended diamond bur (FG 611 C, Intensiv, SA, Lugano, Switzerland) following the cusp inclines. A standardized, centrally positioned MOD cavity was then prepared to a depth of 2.5 mm (1 mm above the CEJ), with a cavity width preserving a 2.5 mm thickness of the buccal and palatal walls, measured at the height of contour [13, 26] (Fig. 3).

#### Endodontic treatment

After cavity preparation, the negative control and other experimental groups underwent standardized endodontic treatment. Root canals were prepared using an endodontic rotary motor system (Endo pace, Woodpecker, China) with 0.04 taper rotary files (Fanta AF blue, Shanghai, China). The canals were irrigated with 2.5% sodium hypochlorite irrigation solution after each rotary file. The canals were then dried using 0.04 taper absorbent paper points (Diadent, Korea) and obturated using a single-cone technique with a 0.04 taper gutta-percha cone (Meta Bio-med, Chungbuk, Korea) and a bio-ceramic sealer (Cera seal, Meta Bio-med, Chungbuk, Korea). Excess gutta-percha was removed to the level of the canal orifice using a heated hand instrument, and the cavity was subsequently cleaned with an alcohol-damped cotton pellet. 

#### Bonding protocol

Following endodontic treatment, selective enamel etching of cavity margins was performed with 37% phosphoric acid etchant (SDI, Australia) for 30 s, followed by rinsing and air-drying. A 2-step universal adhesive (G2 BOND Universal, GC, Tokyo, Japan) was then applied according to the manufacturer’s instructions and light-cured for 20 s through a light-curing unit (Radii Plus, SDI, Australia) with an irradiance output of 1000 mW/cm². The light-curing unit was regularly checked to be fully recharged after every 10 curing cycles throughout the experimental procedures. The canal orifices were sealed with a thin layer of flowable resin composite (Geanial Universal flo, GC Corp, Tokyo, Japan) to standardize the level at which the subsequent core material was applied. 

#### Restorative procedures

**Fig. 4:**
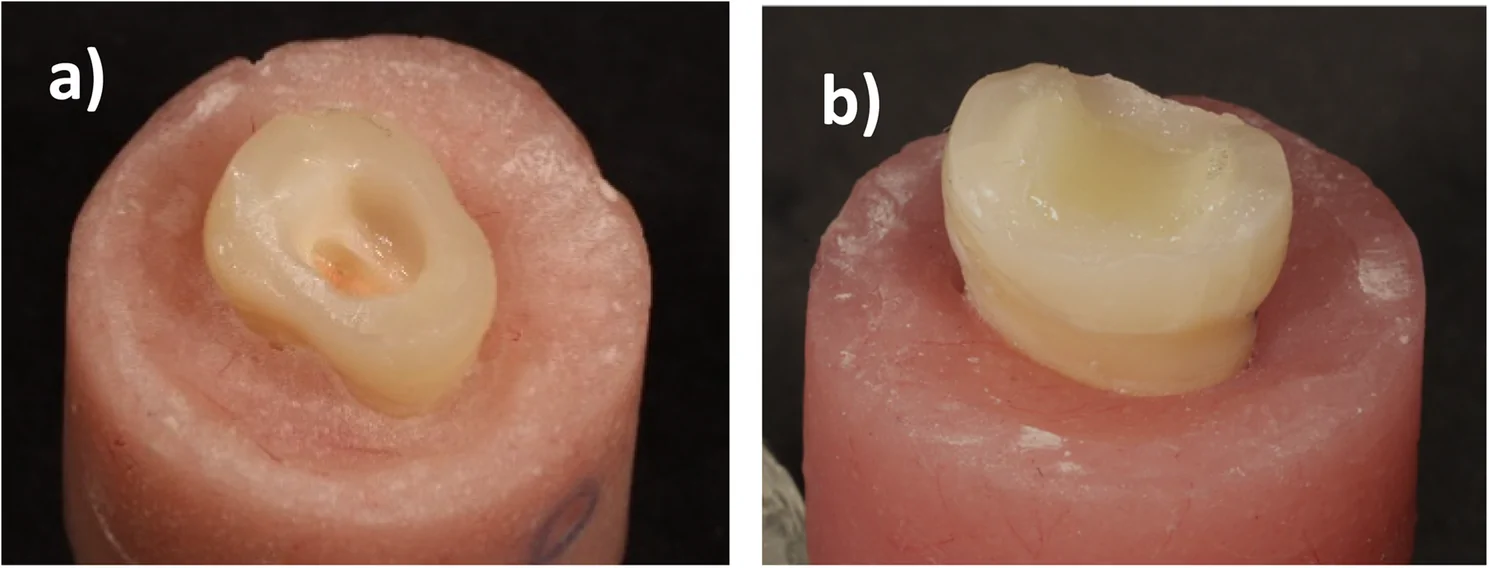
**a** Proximal wall build-up. **b** Core material application

- For the negative control group, MOD cavities were restored using direct oblique incremental packing technique with packable PFC (G-ænial A’CHORD, GC Corp, Tokyo, Japan); each increment was light-cured for 20 seconds.
- For the remaining specimens (*n* = 90), the proximal walls were first built to a thickness of 1.5 mm against the corresponding proximal index using packable PFC (G-ænial A’CHORD, GC Corp, Tokyo, Japan). Each group (*n* = 30) then received bulk filling [27] with one of the 3 core materials up to the level of the internal cavo-surface margin, with an increment thickness of 2.5 mm, followed by light-curing for 20 seconds (Fig. 4).

After that, each group was further subdivided into three subgroups (*n* = 10) according to the type of occlusal overlay restoration: 

#### Direct overlay (D)

**Fig. 5:**
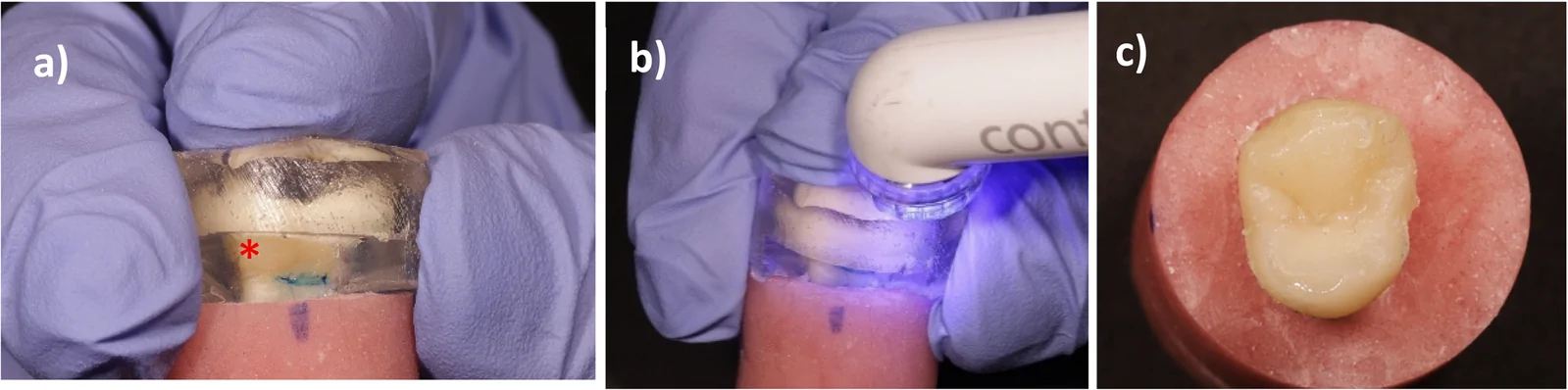
Steps for direct overlay fabrication. **a** Clear silicon index pressed against specimen; asterisk indicates excess material extrusion. **b** Three-finger pressure maintained during light curing through the index. **c** Overlay after removing the clear index

Packable PFC (G-ænial A'CHORD, GC, Tokyo, Japan) was placed into the clear silicone index corresponding to the respective tooth and seated against the core surface. Three-point finger pressure was applied simultaneously from the buccal, occlusal, and palatal aspects and maintained for 10 seconds to ensure adequate material adaptation and allow excess material to flow through the proximal vent of the index. The excess material was removed, and the restoration was light-cured through the silicone index for 20 seconds while maintaining the three-point finger pressure. Subsequent complete curing was performed without the index for 20 seconds on each surface (Fig. 5).

#### Indirect overlay (ID)

**Fig. 6:**
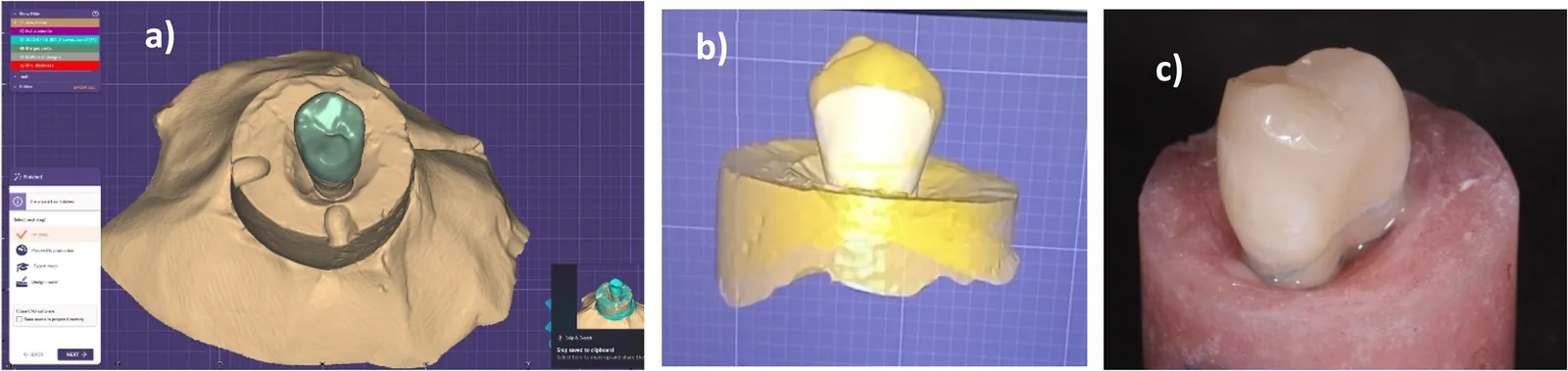
Steps of indirect overlay fabrication. **a** The pre-operative scan record. **b** Adjusting the pre-operative scan onto the preparation. **c** Indirect overlay after cementation

The core surface was finished using a yellow-coded tapered diamond bur (FG 5237 intensiv, Switzerland) and then scanned. The restorations were digitally designed utilizing the pre-operative scan of each corresponding tooth to reproduce its original anatomy. The occlusal thickness was standardized at 2 mm, with a cement space of 0.1 mm. Hybrid CAD/CAM blocks (CERASMART 270, GC, Tokyo, Japan) were wet-milled using a 5-axis milling machine (CORiTec 350i PRO imes-icore, GmbH, Germany) (Fig. 6a, b).

#### Cementation protocol

Cementation was performed according to the manufacturer’s instructions. The intaglio surface was air-abraded with 50 μm aluminum oxide particles (Cobra, Renfert, Hilzingen, Germany) at a pressure of 2 bar, from a distance of 1 cm, for 5 s, followed by rinsing and air-drying. A silane-containing primer (G-Multi Primer, Tokyo, Japan) was then applied, allowed to react for 1 min, and gently air-dried. The prepared tooth surface was air-abraded with 50 μm aluminum oxide particles at a pressure of 2 bar, from a distance of 2 cm, for 5 s. Selective enamel etching was then performed, followed by adhesive application to both the tooth and core surfaces and subsequent light-curing. The restoration was cemented with a universal self-adhesive resin cement (G-CEM ONE, GC, Tokyo, Japan). A standardized load of 1 kg was applied using a custom-made loading device. The resin cement was tack-cured for 1 s, and excess cement was carefully removed. Final light-curing was then performed for 20 s from each surface (Fig. 6c). 

#### Semi-direct overlay (SD)

the core surface was finished using a yellow-coded tapered diamond stone (FG 5237, Intensiv, Switzerland). To standardize the cement space, two sheets of medium-density polytetrafluoroethylene (PTFE; Teflon) tape (Kohinoor, China), each 0.075 mm thick, were carefully adapted to the core surface to provide a cement space of approximately 0.15 mm. A thin film of separating medium was applied to the Teflon tape [6]. The semi-direct overlay was fabricated using packable PFC (G-ænial A’CHORD, GC, Tokyo, Japan), which was placed into the corresponding clear silicone index, seated against the specimen, and light-cured following the same protocol described for the direct overlay group. The separating medium was removed, and the intaglio surface was cleaned with 37% phosphoric acid etchant for 15 s, followed by rinsing and air-drying [28, 29]. Silanization and cementation were subsequently performed using the same protocol described for the indirect overlay group.

All specimens were then polished using diamond-impregnated rubber polishers (Enhance Po-Go, Dentsply Sirona, PA, USA) and stored in distilled water at room temperature for 24 h, before fracture resistance testing.

### Fracture resistance testing

**Fig. 7:**
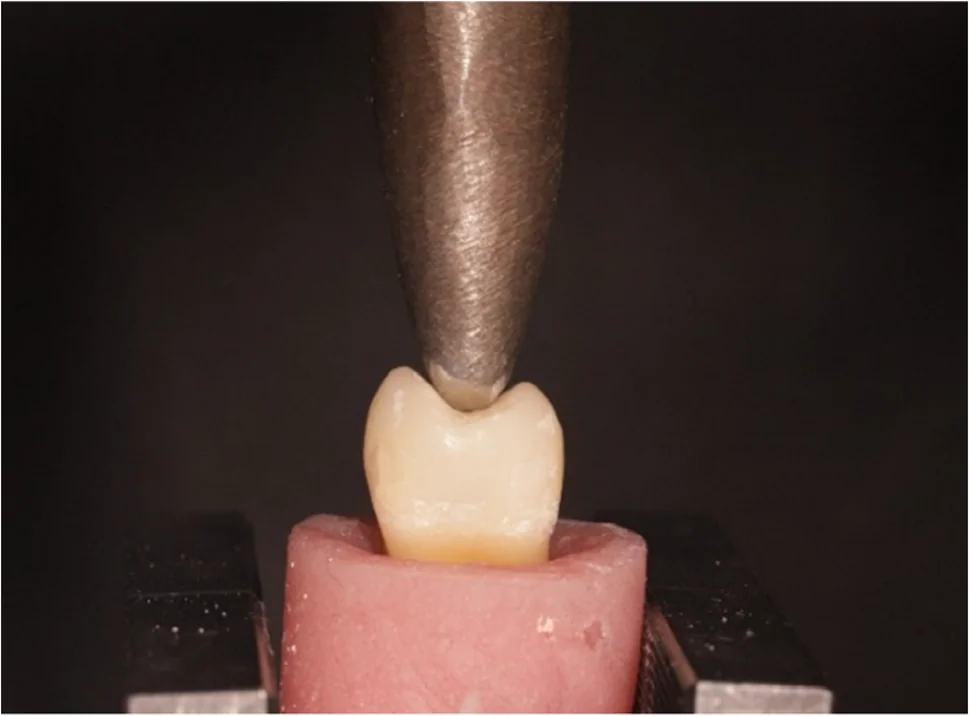
Loading piston adjusted on occlusal surface

Specimens were tested for fracture resistance using a universal testing machine (Instron 3345, Bucks, UK) with a vertical loading piston featuring a 5 mm-diameter rounded tip positioned to contact both cusp inclines (Fig. 7). A crosshead speed of 1 mm/min was applied and the maximum load (*N*) was recorded at the point of an audible signal indicating the cracking and the first noticeable drop in the load-displacement curve.

### Failure mode analysis

**Fig. 8:**
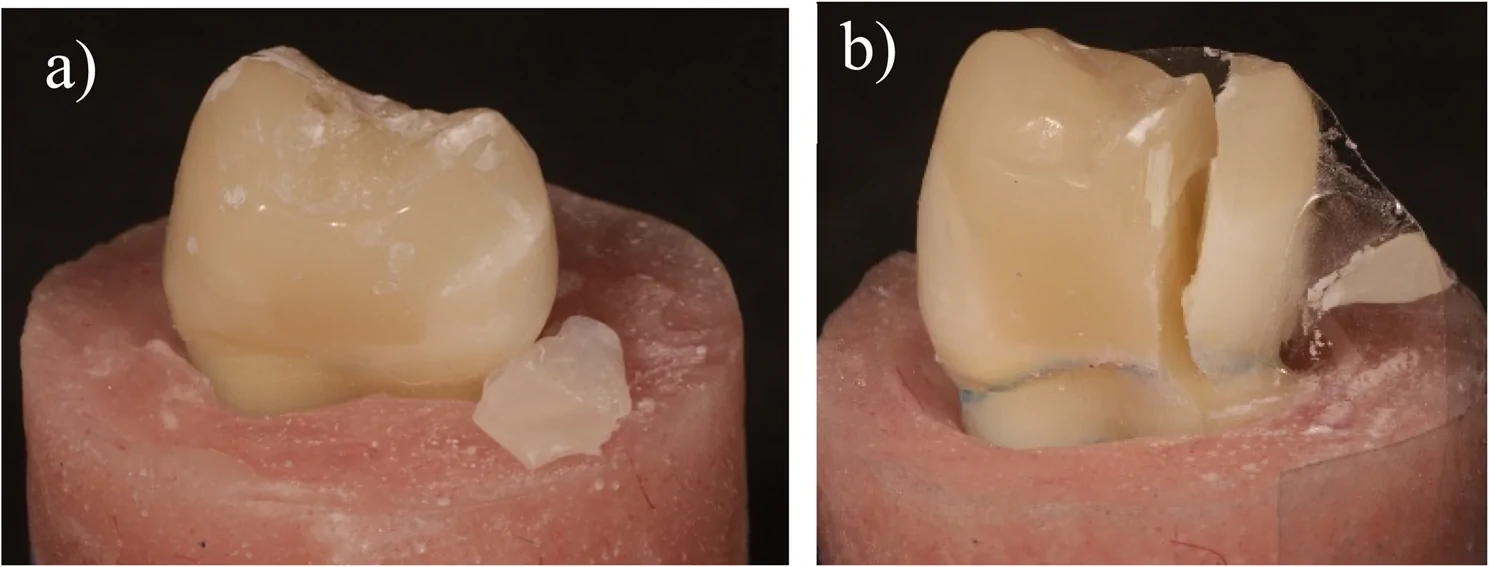
Different failure mode: **a** Favorable fracture. **b** Unfavorable fracture

Fractured specimens were visually examined using macro-photographs obtained with a 100 mm macro lens mounted on a Canon EOS 6D Mark II digital single-lens reflex (DSLR) camera (Canon Inc., Tokyo, Japan) to determine the failure mode. Fractures were classified as favorable or unfavorable fractures [25] (Fig. 8):

-Favorable fracture: Crown fractures extending above or at the CEJ.

-Unfavorable fracture: Crown fracture extending more than 1 mm below the CEJ.

### Statistical analysis

- Fracture load numerical values expressed in Newton (*N*) were presented as mean and standard deviation. The assumptions of normality and homogeneity of variance were assessed using the Shapiro–Wilk and Levene tests, respectively. Data were statistically analyzed for the effect of the two variables (core and overlay materials) interactions using two-way ANOVA. Post hoc comparisons of estimated marginal means were performed using F- and pairwise t-tests with *p*-values adjusted for multiple comparisons using Holm's method.
- The positive and negative control groups were compared using an independent t-test. Comparisons between the experimental groups and the control group were conducted using Dunnett contrasts.
- Categorical data (failure modes) were presented as frequencies and percentages. They were analyzed to assess the effects of different variables using a Firth logistic regression model. Additionally, the control groups were compared using Fisher's exact test.
- The significance level was set at *p* < 0.05 for all tests. Statistical analysis was performed with R statistical analysis software version 4.5.2 for Windows (R Core Team, 2025).

## Results

### Fracture resistance

Significant interactions were observed between the two variables (*p* < 0.001), with a moderate effect size (ω² = 0.24). The results of two-way ANOVA are summarized in Table 2, while detailed group interactions are presented in Table 3.

**Table 2:** Two-way ANOVA of the effect of different study variables and their interactions on fracture resistance (*N*)

Variable	Sum of squares	df	f-value	*p*-value	ω^2^ (95% CI)
Core material	1259189.99	2	8.44	< 0.001*	0.16 (0.03 to 0.30)
Overlay material	489976.31	2	3.29	0.043*	0.05 (0.00 to 0.17)
Core x Overlay material	2197378.64	4	7.37	< 0.001*	0.24 (0.06 to 0.37)

*df* degree of freedom, *CI* confidence interval

asterisks indicate significance (*p* < 0.05)

**Table 3:** Comparisons and summary statistics of fracture resistance (*N*) for different variables

Overlay material	Fracture resistance (*N*) (Mean ± SD)			*p*-value	ω^2^ (95% CI)
	EXP	EXF	GC		
D	1124.61 ± 213.32^Aa^	596.14 ± 171.40^Bc^	879.75 ± 211.27^ABb^	< *0.001	0.16 (0.03 to 0.30)
ID	1140.47 ± 201.45^Ba^	1514.28 ± 330.01^Aa^	1551.52 ± 381.68^Aa^	*0.002	0.13 (0.01 to 0.27)
SD	850.05 ± 251.58^Aa^	1001.16 ± 299.74^Ab^	1018.63 ± 309.11^Ab^	0.351 ns	0.00 (0.00 to 0.02)
*p*-value	0.051 ns	< *0.001	< *0.001		
ω^2^ (95% CI)	0.05 (0.00 to 0.17)	0.39 (0.22 to 0.53)	0.28 (0.12 to 0.43)		

Values with different uppercase and lowercase superscripts within the same row and column, respectively, are significantly different

*ns* not significant

Asterisks indicate significance

Regarding different interactions, EXP demonstrated the highest fracture resistance within the direct overlay group (1124.61 ± 213.32 N). It showed no significant difference in fracture resistance within the other overlay restoration groups; indirect (1140.47 ± 201.45 N) and semi-direct (850.05 ± 251.58 N) groups (*p* = 0.051, ω^2^ = 0.05).

GC and EXF demonstrated significant differences in fracture resistance across the 3 overlay restorations (*p* < 0.001, ω^2^ = 0.28 and *p* < 0.001, ω^2^ = 0.39, respectively). The highest values for both materials were observed in the indirect overlay group. EXFID and GCID showed significant differences from both control groups (Table 4).

GC and EXF showed no significant difference in fracture resistance compared with EXP in the semi-direct group (*p* = 0.351, ω^2^ = 0.00). EXF showed significantly lower fracture resistance than EXP in the direct group (596.14 ± 171.40 N), whereas GC did not show a significant difference in fracture resistance to either EXP or EXF in the same group (879.75 ± 211.27 N) (Table 3).

No significant difference was found between the positive and negative control groups (*p* = 0.352, Hedge’s g = 0.42). Compared with the negative control, EXPD (1124.61 ± 213.32 N, *p* = 0.044) showed a significant difference along with the relevant indirect overlay subgroups (Table 4). 

**Table 4:** Comparisons and summary statistics of fracture resistance (*N*) between the control groups and experimental groups

Groups	Fracture resistance (*N*) (Mean ± SD)	Compared with Positive control		Compared with Negative control	
		*p*-value	Hedge’s g (95% CI)	*p*-value	Hedge’s g (95% CI)
Positive control	855.29 ± 157.92	—	—	0.352 ns	0.42 (-0.46 to 1.29)
Negative control	786.09 ± 157.03	0.352 ns	0.42 (-0.46 to 1.29)	—	—
EXPD	1124.61 ± 213.32	0.187 ns	-1.37 (-2.35 to -0.35)	0.044*	-1.74 (-2.76 to -0.69)
EXFD	596.14 ± 171.40	0.220 ns	1.50 (0.46 to 2.50)	0.529 ns	1.11 (0.16 to 2.03)
GCD	879.75 ± 211.27	1 ns	-0.12 (-0.98 to 0.74)	0.975 ns	-0.48 (-1.33 to 0.38)
EXPID	1140.47 ± 201.45	0.126 ns	-1.49 (-2.47 to -0.48)	0.025*	-1.88 (-2.90 to -0.82)
EXFID	1514.28 ± 330.01	< 0.001*	-2.39 (-3.54 to -1.20)	< 0.001*	-2.70 (-3.89 to -1.47)
GCID	1551.52 ± 381.68	< 0.001*	-2.23 (-3.35 to -1.07)	< 0.001*	-2.51 (-3.66 to -1.32)
EXPSD	850.05 ± 251.58	1 ns	0.02 (-0.86 to 0.90)	0.998 ns	-0.30 (-1.16 to 0.57)
EXFSD	1001.16 ± 299.74	0.805 ns	-0.58 (-1.47 to 0.33)	0.388 ns	-0.87 (-1.77 to 0.05)
GCSD	1018.63 ± 309.11	0.682 ns	-0.62 (-1.50 to 0.27)	0.276 ns	-0.91 (-1.79 to -0.01)

*CI* confidence interval, *ns* not significant

asterisks indicate significance (*p* < 0.05)

### Failure mode analysis

No significant differences were observed in failure modes among the experimental groups. Unfavorable fractures predominated across the experimental groups (Table 5), while a significant difference was only observed between the positive and negative control groups (Table 6).

**Table 5:** Illustrates the percentage of favorable and unfavorable failures among different groups

Overlay material	Failure mode	*n* (%)			*p*-value	ω^2^ (95% CI)
		EXP	EXF	GC		
D	Favorable	1 (10.00%)	1 (10.00%)	0 (0.00%)	0.769 ns	0.08 (0.00 to 0.24)
	Unfavorable	9 (90.00%)	9 (90.00%)	10 (100.00%)		
ID	Favorable	2 (20.00%)	1 (10.00%)	2 (20.00%)	0.843 ns	0.06 (0.00 to 0.21)
	Unfavorable	8 (80.00%)	9 (90.00%)	8 (80.00%)		
SD	Favorable	3 (30.00%)	1 (10.00%)	1 (10.00%)	0.515 ns	0.12 (0.00 to 0.30)
	Unfavorable	7 (70.00%)	9 (90.00%)	9 (90.00%)		
*p*-value		0.636ns	1ns	0.547ns		
ω^2^ (95% CI)		0.10 (0.00 to 0.27)	0.00 (0.00 to 0.00)	0.12 (0.00 to 0.29)		

*CI* confidence interval, *ns* not significant

**Table 6:** Comparisons and summary statistics of failure modes for different control groups

Failure mode	*n* (%)		*p*-value	Odds ratio (95% CI)
	**Positive control**	**Negative control**		
Favorable	7 (70.00%)	1 (10.00%)	0.020*	21.00 (1.78 to 248.11)
Unfavorable	3 (30.00%)	9 (90.00%)		

*CI* confidence interval

asterisks indicate significance (*p* < 0.05)

## Discussion

This study investigated the effect of different core and overlay materials on the fracture resistance of bi-layered restorations in compromised endodontically treated maxillary premolars, aiming to identify optimal restorative combinations. It was conducted on maxillary premolars due to their increased susceptibility to unfavorable crown and vertical root fractures compared to molars [30]. The first, second, and third null hypotheses were rejected, as core material, occlusal overlay restoration type, and their interaction significantly affected the fracture resistance of the bi-layered restorations.

Among the tested bi-layered restorations, the indirect overlay group exhibited the highest fracture resistance values, which were significantly higher than the negative control group. This superior performance may be attributed to the enhanced mechanical properties of the CAD/CAM hybrid restoration (CERASMART 270), which may provide favorable mechanical performance than conventional direct PFC materials [6, 25]. Moreover, the 2-mm thick overlay design might contribute to enhanced stiffness and greater stress absorption within the restorative structure [1, 15, 31], regardless of the type of underlying core material. The presence of multiple interfaces, including the adhesive and resin cement layers, may also have influenced stress transmission from the overlay restoration to the core [1, 9], thereby limiting the reinforcing effect of the underlying core material and allowing the overlay to function relatively independently.

The semi-direct overlay group demonstrated lower fracture resistance than the indirect overlay group. This may be attributed to the use of a direct composite material without additional polymerization techniques, which may have likely limited the enhancement of the mechanical properties [6]. Additionally, the manual procedure was more technique-sensitive when compared to the digital workflow. All core material subgroups showed comparable results, with no statistically significant difference among them. This uniformity may indicate a reduced reinforcing effect of the different core materials, potentially due to the buffering effect of the resin cement interface [2].

The direct overlay group exhibited the greatest variability in fracture resistance among the experimental groups, with marked discrepancy in the fracture resistance across the core materials, highlighting the strong influence of the underlying core material on reinforcing the direct overlay restorations [32]. This may be attributed to the presence of the oxygen-inhibited layer (OIL), which may promote optimal interfacial adhesion, structural continuity, and efficient stress transfer [9], particularly when adequate material adaptation is achieved. Consequently, promoting the underlying core material to express its structural properties and contributes to the load-bearing capacity of the restorative system.

Regarding different core materials, everX Posterior (EXP) demonstrated consistently high fracture resistance among the experimental overlay groups. This behavior may be attributed to its high fiber and filler loading (61–75 wt%), which contributes to its relatively high flexural modulus (12.6 ± 2.8 GPa) [33]. Furthermore, the short randomly oriented fibers allow a high fiber volume fraction, while implementing the appropriate critical fiber length may facilitate absorption of a greater amount of stress along the fiber length, thereby contributing to the load-bearing capacity of the material under compressive stresses transmitted from the occlusal overlay restoration [34, 35].

everX Flow (EXF) demonstrated significant variable performance across the experimental overlay groups. The highest fracture resistance was recorded when layered with the indirect overlay group, whereas the lowest value was observed with the direct overlay group. These findings differ from a study conducted by Frater et al. [25], who concluded no significant difference in fatigue strength between direct and indirect overlays when a flowable SFRC substructure was used as a 6-mm thick post and core material. In their study, EXF was subjected to fatigue strength testing, which relies on cyclic loading at relatively low force magnitudes and may favor materials with a low modulus of elasticity [36]. EXF may offer ease of application and favorable handling properties, but its shorter fibers (140 μm), reduced glass filler content, and UDMA-based matrix contribute to a relatively lower modulus of elasticity (9.0 ± 0.7 GPa) [14, 33]. This may have contributed to its limited mechanical support when used beneath the 2 mm-thick direct overlay. However, this limitation may have been less apparent when EXF was combined with the 2 mm-thick indirect overlays.

The same rationale may also explain the comparable performance of GRADIA CORE material (GC) between the direct and indirect overlay groups. In the direct overlay group, GC demonstrated intermediate fracture resistance with no significant difference from either EXP or EXF; however, in the indirect overlay group, it demonstrated higher fracture resistance comparable to that of EXF. This may be attributed to the interfacial characteristics of flowable materials, which may provide better adaptation and bonding efficacy, particularly in the indirect restorations where multiple interfaces are present [37].

Several interactions demonstrated consistent mechanical behavior across the experimental groups, whereas other bi-layered restorations exhibited a notable improvement in fracture resistance. Among the direct bi-layered restorations, EXPD demonstrated the highest fracture resistance. Additionally, EXP was the only core material in the direct overlay group to show a significant difference (*p* = 0.044) from the negative control group. Despite its limited core thickness (2.5 ± 0.5 mm), which falls below the recommended reinforcing thickness relative to the overlying restoration [15], its inherent structural properties may have contributed to reinforcement of the direct occlusal overlay restoration, in contrast to its flowable counterpart.

However, in the indirect group, EXPID demonstrated significantly lower fracture resistance than EXFID and GCID. This may be attributed to the surface topography of the core-cement interface, which can be influenced by the surface treatment performed. Different surface treatments can produce varying degrees of surface roughness, which may affect resin cement wettability, interfacial adhesion [38], and the overall mechanical behavior of the bi-layered restoration [39, 40]. EXP may exhibit greater surface roughness after performing finishing and air abrasion procedures compared with other bulk-fill composite materials [34]. Air-abrasion of the EXP surface can result in scratches, exposed fibers, and fiber loss, creating crater-like depressions on the surface [41]. A rough surface can also create localized stress concentration points along the interface, potentially contributing to reduced fracture resistance, whereas a relatively flat and intact interface may be less prone to develop such weak points [39]. EXP contains short fibers with a diameter of approximately 15 μm [42], whereas EXF utilizes smaller fibers with a diameter of approximately 6 μm [43]. This difference may partly explain the observed significant difference in fracture resistance, potentially due to differences in the surface roughness profiles acquired during the preparation procedures. This interpretation is consistent with the findings of Lassila et al. [44], who compared the surface roughness of different packable and flowable SFRCs after laboratory polishing with 4000-grit abrasive paper. They reported a significant difference in surface roughness between everX Posterior (0.42 μm) and everX Flow (0.26 μm). It is plausible that the use of a finishing stone with a surface roughness of approximately 15 μm [45], together with air abrasion using 50 μm aluminum oxide particles, may have contributed to greater surface roughness of EXP than that of flowable core materials such as EXF and GC [46].

Regarding failure mode analysis, no significant difference was found among the groups, leading to acceptance of the fourth null hypothesis. The predominant mode of failure was unfavorable bulk fracture apical to the CEJ. This finding is consistent with other studies [15, 18, 25] and may be related to the 2 mm-thick overlay restoration, which may allow the overlay to withstand higher stresses and increase the stored energy generated upon loading [1], potentially causing the crack front to propagate through the material in one direction with limited deviation [18]. In contrast, other studies reported a favorable failure mode of the coronal restoration when reinforced with SFRC [22]. These findings have been attributed to the ability of glass fibers to arrest or deflect crack propagation, and the ability of indirect overlay restorations to absorb stress, thereby promoting a more favorable failure mode [47].

The predominance of unfavorable fractures in our study may also be related to the lack of periodontal ligament (PDL) simulation, which could influence fracture patterns [48]. However, PDL simulation was not incorporated in the static load testing due to concerns regarding the functional validation, experimental reliability, and standardization of currently available PDL simulation methods [49]. Its inclusion may introduce uncontrolled variation and potentially influence the study’s outcomes.

Several of the tested bi-layered structures exhibited fracture resistance values comparable to those of the positive control group. Furthermore, the average normal biting force has been reported to be approximately 332.5 N, while clenching forces may reach up to 800 N [30]. However, these values represent static load-to-fracture and do not replicate cyclic loading or any environmental changes occurring in the oral cavity.

The present study aimed to provide high-quality and concise initial data regarding the immediate fracture resistance of different bi-layered restorations, using standardized tooth selection, cavity preparation, and testing methods. Aging methods were not incorporated in this study to allow direct comparison of the intrinsic mechanical performance of the experimental groups without additional influence of aging-related degradation [50, 51]. Vertical static load-to-fracture testing was performed instead of off-axis loading to promote a more uniform stress distribution among the specimen’s components [52]. Therefore, the present findings should be interpreted with caution, and future studies incorporating artificial aging protocols, off-axis loading, and cyclic fatigue testing are warranted to closely simulate the intraoral environment and further establish the clinical relevance of the findings.

## Conclusions

Within the limitations of this study, it can be concluded that the fracture resistance of bi-layered restorations is influenced by the interaction between core and occlusal overlay material across different restorative approaches (direct, indirect, semi-direct). Packable SFRC (everX Posterior) offers a reinforcing effect in direct bi-layered restorations of root canal-treated maxillary premolars. In contrast, flowable SFRC and conventional dual-cured core material demonstrated higher fracture resistance than packable SFRC under indirect overlay restorations. The 3 core materials demonstrated comparable fracture resistance within the semi-direct overlay restorations. A 2 mm-thick indirect hybrid CAD/CAM overlay demonstrated high fracture resistance comparable to that of the direct bi-layered restoration using everX Posterior. Therefore, the indirect hybrid CAD/CAM overlay should not be interpreted as universally superior to all direct restorative approaches.

## Data Availability

Availability of data and materials: Available upon request from the corresponding author.

## Abbreviations

MOD: Mesio-occluso-distal
PFC: Particulate-filled composite
SFRC: Short fiber-reinforced composite
OIL: Oxygen-Inhibited layer
CEJ: Cemento-enamel junction
PDL: Periodontal Ligament
PTFE: Polytetrafluoroethylene
EXF: everX Flow
GC: GRADIA CORE
EXP: everX Posterior
D: Direct
ID: Indirect
SD: Semi-direct
NC: Negative Control
